# NG2 cells response to axonal alteration in the spinal cord white matter in mice with genetic disruption of neurofilament light subunit expression

**DOI:** 10.1186/1750-1326-3-18

**Published:** 2008-10-28

**Authors:** Ya Jun Wu, Ya Fang Tang, Zhi Cheng Xiao, Zhen Min Bao, Bei Ping He

**Affiliations:** 1 Division of Life Science and Technology, Ocean University of China, Qingdao, Shandong, PR China; 2 Department of Anatomy, Yong Loo Lin School of Medicine, National University of Singapore, Singapore; 3 Department of Clinical Research, Singapore General Hospital, Singapore; 4 Centre for Life Sciences, National University of Singapore, Singapore

## Abstract

**Background:**

Chondroitin sulphate proteoglycan (NG2) expressing cells, morphologically characterized by multi-branched processes and small cell bodies, are the 4^th ^commonest cell population of non-neuronal cell type in the central nervous system (CNS). They can interact with nodes of Ranvier, receive synaptic input, generate action potential and respond to some pathological stimuli, but the function of the cells is still unclear. We assumed the NG2 cells may play an active role in neuropathogenesis and aimed to determine if NG2 cells could sense and response to the alterations in the axonal contents caused by disruption of neurofilament light subunit (NFL) expression.

**Results:**

In the early neuropathological development stage, our study showed that the diameter of axons of upper motor neurons of NFL-/- mice decreased significantly while the thickness of their myelin sheath increased remarkably. Although there was an obvious morphological distortion in axons with occasionally partial demyelination, no obvious changes in expression of myelin proteins was detected. Parallel to these changes in the axons and their myelination, the processes of NG2 cells were disconnected from the nodes of Ranvier and extended further, suggesting that these cells in the spinal cord white matter could sense the alteration in axonal contents caused by disruption of NFL expression before astrocytic and microglial activation.

**Conclusion:**

The structural configuration determined by the NFL gene may be important for maintenance of normal morphology of myelinated axons. The NG2 cells might serve as an early sensor for the delivery of information from impaired neurons to the local environment.

## Background

Neurodegenerative diseases are the main causes for disability, dementia, and death in elderly people. Two common signs of neurodegenerative diseases observed from clinically characterized autopsy tissues at the terminal stage of the diseases are neuronal cell death and glial cell activation. However, the causative relations between these two phenomena are still not fully understood, especially at the early stage of neuropathogenesis.

Previous studies have reported that abnormal neurofilament aggregates are often associated with decreases in the level of NFL mRNA, for instance, more than 70% downregulation of NFL mRNA was detected in degenerating neurons of amyotrophic lateral sclerosis (ALS) [1,2]. Therefore, in our previous study, we adopted a mouse model for ALS. In this model, neurodegeneration is initiated in neurons after disruption of NFL expression. The NFL-/- mice will develop abnormal protein accumulation in neuronal perikarya and proximal axons, a common phenomenon in neurodegenerative diseases, without obvious signs of motor dysfunction in the early stage. They thus serve as a model for investigation of the temporal relationship between the neuronal aggregates and glial activation (another common phenomenon in neurodegeneration diseases) [3]. Three developmental stages of neurodegeneration in the spinal cord of this animal model have been classified. In the first stage, neurofilament heavy subunit (NFH) is redistributed and accumulates in the neuronal perikarya and proximal axons of the spinal cord, together with inhibited expression of the β-subunit (CD11b) of complement receptor type 3 in microglial cells and retarded microglial transformation in response to axotomy [3]. In the second stage, at the age of 6 months, the number of Iba-1 positive microglia increases [4] but not CD11b positive microglia [3] and the number of aggregate-bearing neurons begins to decline [4]. In the last stage, at about 10 months of age, the number of motor neurons decreases with a significant increase in astrocyte numbers [4].

In this current study, we examined the first stage of pathological development in NFL-/- mice, focusing on NG2 cells (a glial cell type with unknown function) in the white matter of lumbar spinal cord segments where axons of upper motor neurons can be found. Based on the hypothesis that neurofilament (NF) redistribution in the upper motor neurons may signal to glial cells in the spinal cord white matter and thereby contribute to the pathogenesis of lower motor neurons in ALS, we explored if NG2 cells in the spinal cord would sense and respond to the pathological alterations in upper motor neurons before the onset of significant motor neuron death.

In the CNS, the NG2 expressing cells are morphologically characterized by multi-branched processes and small cell bodies. They are in contact with nodes of Ranvier, receive synaptic input, and generate action potential [5-8]. It has been proven that rapid signal exchanges exist between the neuronal axons and NG2 cells [9]. However, it is not known if NG2 cells could respond to pathological information from NFL-/- axons in the earlier stage of neuropathogenesis during which microglia are inhibited [3].

The role of NG2 cells in neuronal networks of adult brain is not yet known. Studies have shown that NG2 cells proliferate in response to loss of myelin or oligodendrocytes, as seen in the white matter of spinal cord with genetic myelination defects or induced demyelination [10,11]. Although there is no evidence showing that NG2 cells could directly contribute to remyelination, some NG2 cells have been found to positively express CNPase in the demyelination model [11], suggesting an ability of NG2 cells to differentiate into oligodendrocytes in response to demyelination. However, the similarity in cell numbers and ubiquitous distribution in the brain parenchyma between NG2 cells and astrocytes, microglia or oligodendrocytes in the adult CNS [12-15], have cast doubt on the necessity for having such a large reservoir of progenitor cells in the brain. In various brain injury models without obvious demyelization, the number of NG2 cells adjacent to the damage sites increases with up-regulated expression of NG2 molecules and the cells become hypertrophic [16,17], which suggest that NG2 cells are able to sense other stimulation besides demyelination and therefore they may serve important functions in the brain.

In this study, we provide evidence of the ability of NG2 cells to respond to alterations in the axons of NFL-/- neurons even before astrocytic and microglial activation, which suggest that NG2 cells may serve as an early sensor in neuropathological disorders.

## Results

### 1. NFH protein diminished from neuronal processes and NFH protein aggregates formed in the brain cortex of NFL-/- mice

**Figure 1:**
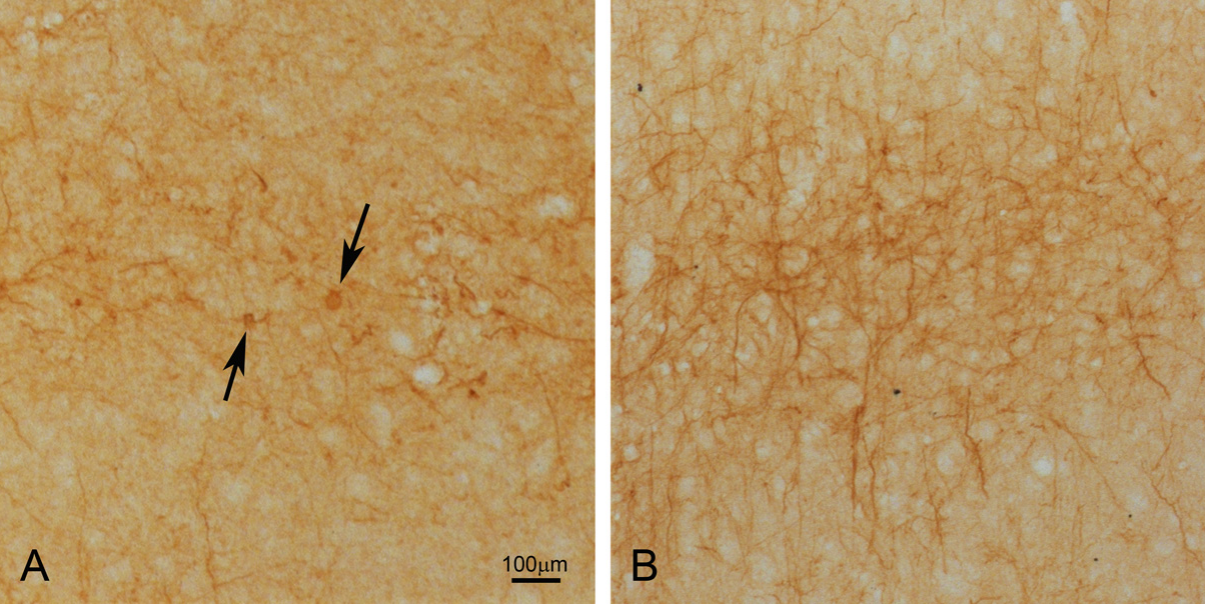
**NFH immunohistochemistry staining of brain cortex**. In the wild type mouse, there were many NFH positive neuronal processes in the lamina V of the sensorimotor cortex (B). However, in the 2 month old NFL-/- mouse, the NFH positive neuronal processes were significantly reduced in number (A) and NFH protein accumulated as aggregates (A, arrows).

Similar to our previous observation [3], in the deep lamina V layer of the sensorimotor cortex of the brain, redistribution of NFH was evident by a reduction of NFH in neuronal processes of neurons, resulting in fewer NFH positive neuronal processes in NFL-/- mice (Fig. 1A) in comparison to the wild type mice (Fig. 1B). Protein aggregates were observed in the regions of the brain cortex of the NFL-/- mice.

### 2. CD11b downregulation in the spinal cord white matter of NFL-/- mice

**Figure 2:**
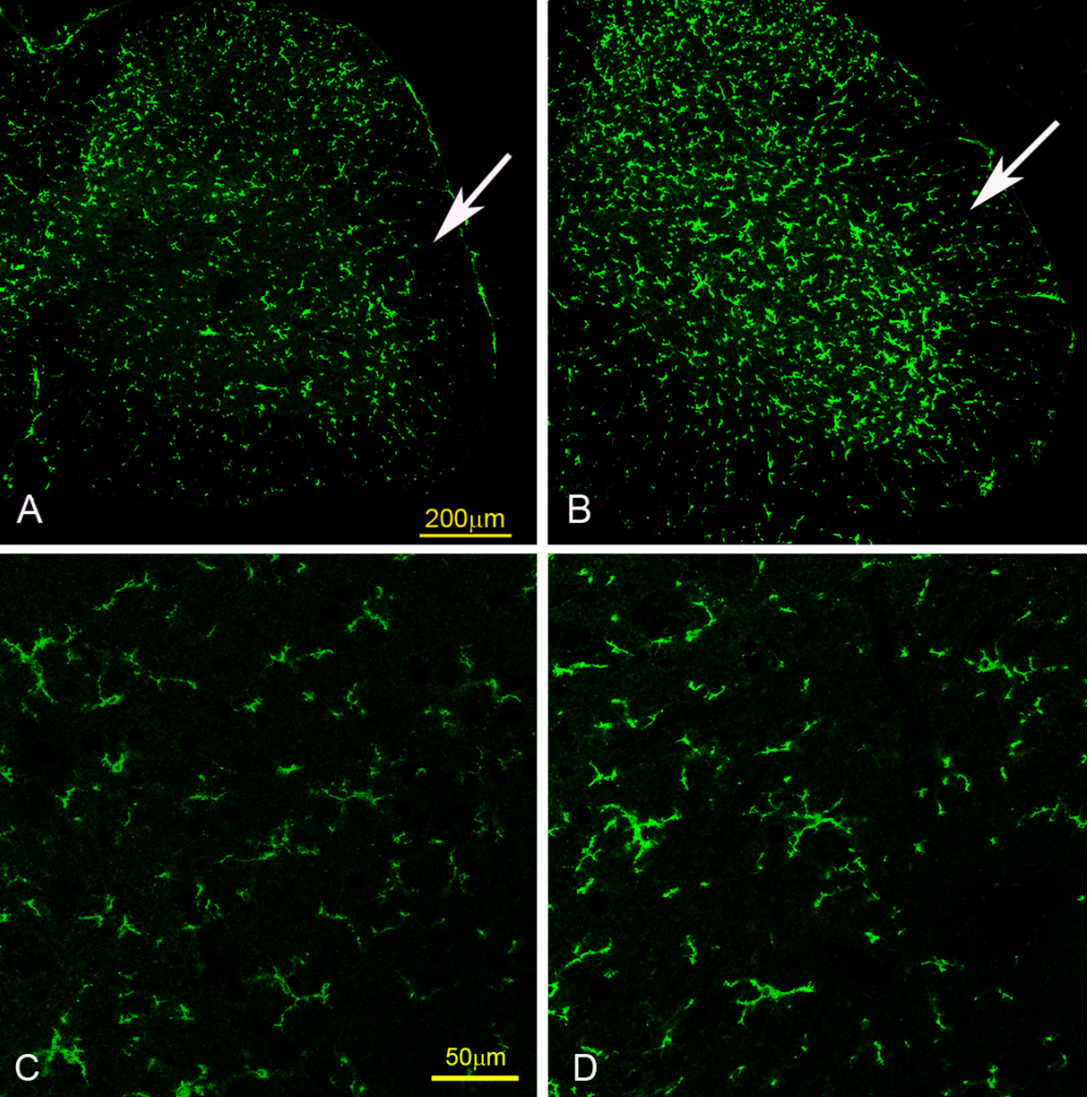
**CD11b staining of spinal cord**. In general, less fluorescent intensity of CD11b immunohistochemistry staining was observed in the spinal cord of NFL-/- mice (A, C) compared to that of wild type mice (B, D), especially in the white matter, at both age of 2 and 6 months. The arrows (A, B) pointed regions are the white matter containing the corticospinal tract in the lateral funiculus of the spinal cord of 2 month old mice (C and D are higher magnification photos taken from arrows pointed areas). Bar: 200 μm in A and B; 50 μm in C and D.

Significant down regulation in CD11b expression in both mRNA and protein levels in the grey matter of lumbar segments of the spinal cord in NFL-/- mice have been demonstrated in our early experiments [3]. In the current study, an obviously lesser immunofluorescent intensity was observed in the white matter containing corticospinal tract (fig. 2, arrows in A and B; C and D higher magnification) of NFL-/- mice (A, C), in comparison against that in the wild type mice (B, D), suggesting a downregulation of CD11b expression in NFL-/- mice.

### 3. Evidence of axonal abnormalities

Our results have indicated that there were some structural abnormalities in axons in the white matter region containing corticospinal tract in the lumbar segments of the spinal cord of NFL-/- mice at the age of 2 and 6 months when there is no obvious sign of activation of astrocytes but inhibition of microglia is observed.

#### Reduction in diameters of myelinated axons and increase in thickness of myelin sheath

**Figure 3:**
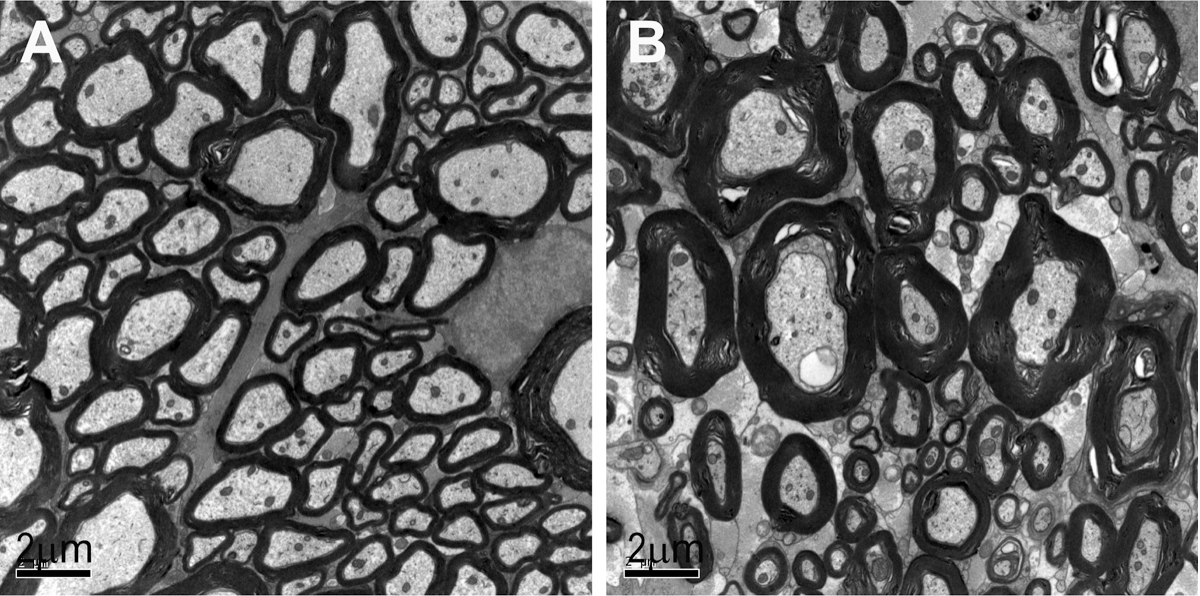
**Electron micrographs of myelinated axons**. Electron micrographs show myelinated axons in the white matter region containing corticospinal tract in wild type (A) and NFL-/- (B) mice at the age of 2 months. Note the myelin thickness increased in NFL-/- mice (B).

**Figure 4:**
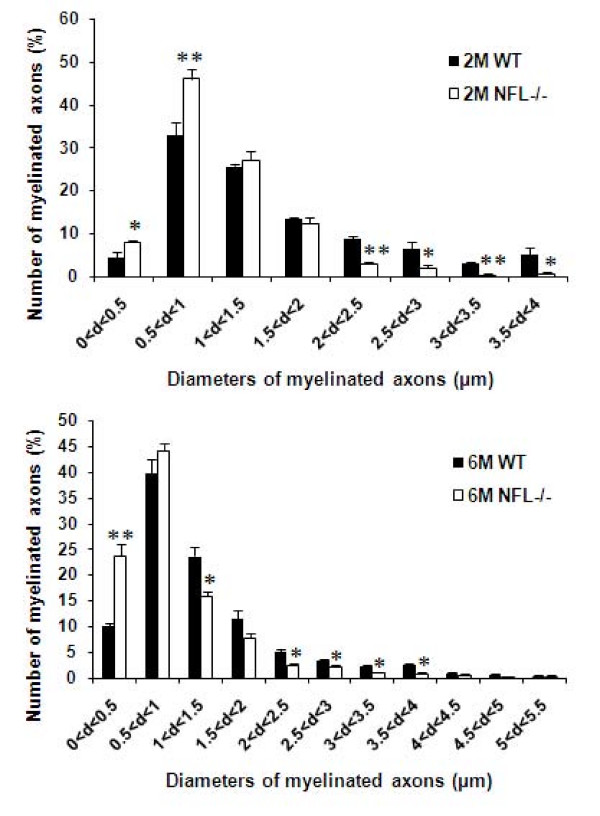
**Frequency distribution of myelinated axons according to their diameters**. Diameters of all myelinated axons were measured in the region containing the corticospinal tract at the lumbar segments of 2- and 6-month-old NFL-/- (N = 3 animals per age group) and control animals (N = 3 animals per age group). Data is presented based on diameters of all axons. Note the marked shift in distribution of axonal diameters towards smaller fibres. (*p < 0.05, **p < 0.01).

In transverse sections of the spinal cord, a significant decrease in axonal diameter and increase in the thickness of myelin sheath were observed in NFL-/- mice (Fig. 3B), in comparison with those from wild type mice (Fig. 3A). Examination of the frequency distribution of axonal diameters in normal and NFL-/- mice (Fig. 4) revealed a shift in diameters of myelinated axons towards smaller sizes compared against wild type mice, suggesting that the absence of NFL subunit results in a reduction in axonal size in the spinal cord white matter.

**Figure 5:**
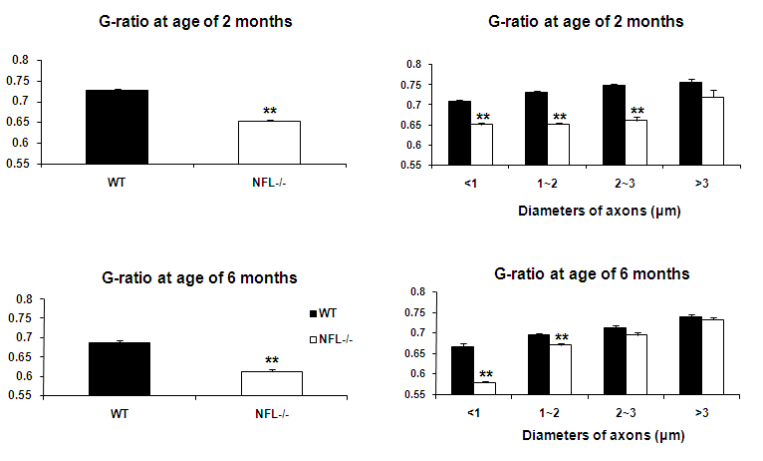
**G-ratio of myelinated axons**. Significant decreases in the G-ratio were measured in NFL-/- mice at both ages of 2 and 6 months (N = 3 animals per age group) in comparison to wild type control (N = 3 animals per age group). However, the decreases were significant only in those axons with smaller diameters in NFL-/- mice. (**p < 0.01).

**Figure 6:**
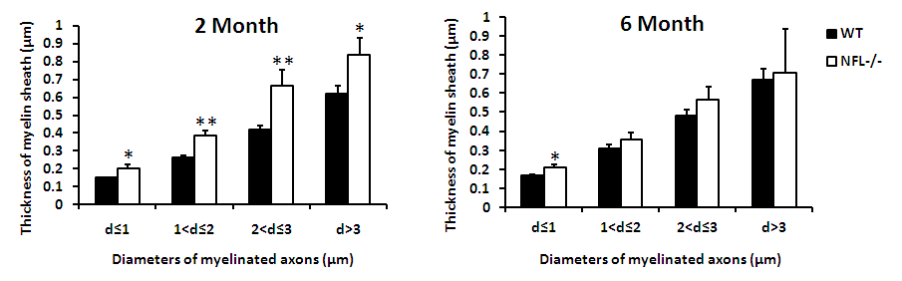
**Thickness of myelin sheath**. The thicknesses of myelin sheath of axons of NFL-/- mice (N = 3 animals per age group) increased significantly in comparison to that of wild type controls (N = 3 animals per age group). (*p<0.05;  **<0.01).

To investigate the relationship between myelin sheath thickness and axonal diameter, the diameters of axon proper (d) and the outer diameter of the myelinated fibre (D) were measured and the G-ratio (d/D) calculated in 2- and 6-month old mice. There was a significant reduction in the G-ratio in NFL-/- mice (Fig. 5), suggesting an increase in the thickness of myelin sheaths and/or decrease in the diameter of axons. The change in the G-ratio in NFL-/- mice was more obvious in the axons with smaller diameters (Fig. 5). Significant increase in the thickness of axonal myelin was observed in the axons of white matter in the spinal cord of NLF-/- mice (Fig. 6). This increase was noted in all ranges of axonal diameters.

#### Increase in number of unloading loops

**Figure 7:**
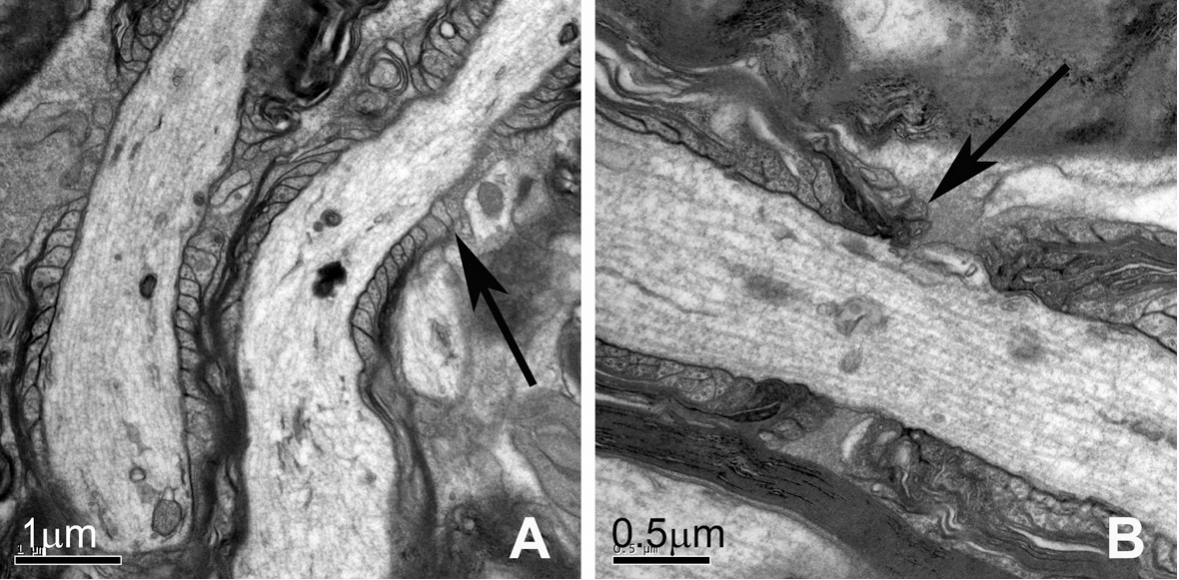
**Electron micrographs of the node of Ranvier**. Electron micrographs show the node of Ranvier in the white matter region containing the corticospinal tract in wild type (A) and NFL-/- (B) mice at the age of 2 months. Note there were more unloading loops (B, arrow), which did not contact with the axonal membrane, in NFL-/- mice. The loading loops in wild type mice are shown in A (arrow).

**Figure 8:**
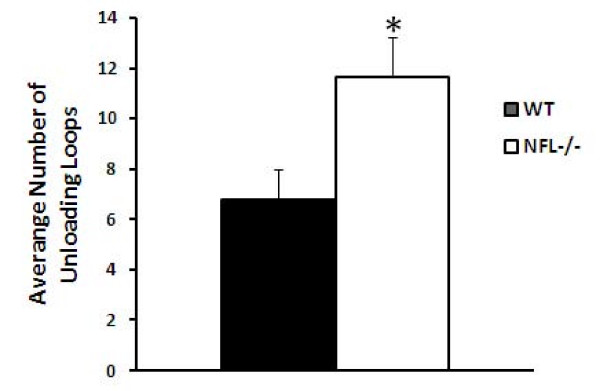
**Numbers of loops at the node of Ranvier**. The number of unloading loops, which have yet to contact with axonal membrane, was significantly increased in the NFL-/- mice near the age of 2 months.

At the node of Ranvier, most myelin loops were found to contact with the axonal membrane (arrow in Fig. 7A), which are referred as loading loops. There were more unloading loops (arrow in Fig. 7B) which are not interacting with the axonal membrane in the NFL-/- mice. Counting the numbers of loops within a 1-μm length of paranodes showed that unloading loops at the node of Ranvier in NFL-/- mice increased significantly in 2-month old mice (Fig. 8), but returned to a normal range at 6 months of age.

#### Distortion and degeneration in myelinated axons

**Figure 9:**
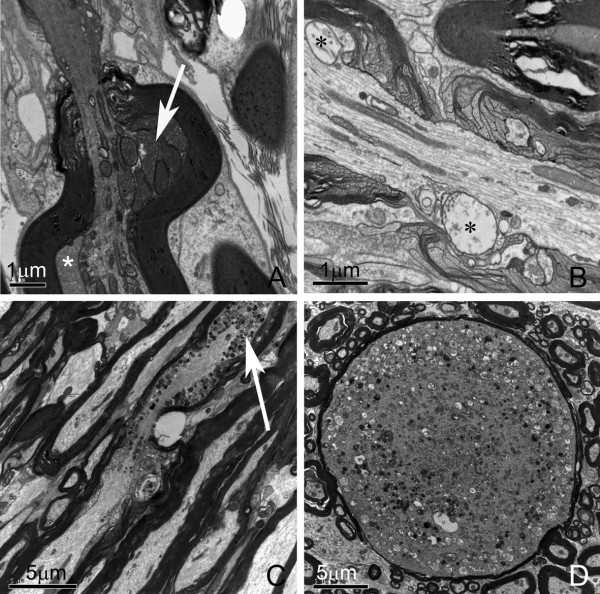
**Electron micrographs of myelinated axons in NFL-/- mice**. Obvious twist of myelinated axons can be observed (A). Accumulation of mitochondria in the axon (A, arrow) and accumulation of amorphological cytoplasm materials between myelin sheath and axon (A, asterisk) were found near the node of Ranvier. Some mitochondria became enlarged and vacuolated (B, asterisks). An obvious accumulation of mitochondria could be detected in certain region of axons (C, arrow), which may lead to a giant axonal sphere stuffed with mitochondria (D). Note the large number of mitochondria that were vacuolated. (A and B from mice at age of 2 months, C and D from mice at age of 6 months).

Further observation using an electron microscope showed that the absence of NFL subunit in neurons resulted in profound changes in axons before the appearance of astrocytic and microglial activation and dysfunctions in the NFL-/- mice. Obvious twist of myelinated axons was present in NFL-/- mice, which might be a result of accumulation of mitochondria in particular regions of the axon (Fig. 9A, arrow) or accumulation of amorphological cytoplasmic material in between the myelin sheath and axon (A, asterisk). Some mitochondria became vacuolated (Fig. 9B, asterisks). Drastic accumulation of mitochondria was occasionally noted in a few axons (Fig. 9C, arrow), which could lead to a giant axonal sphere (Fig. 9D).

#### Loss of regular arrangement of Na^+ ^and K^+ ^channel

**Figure 10:**
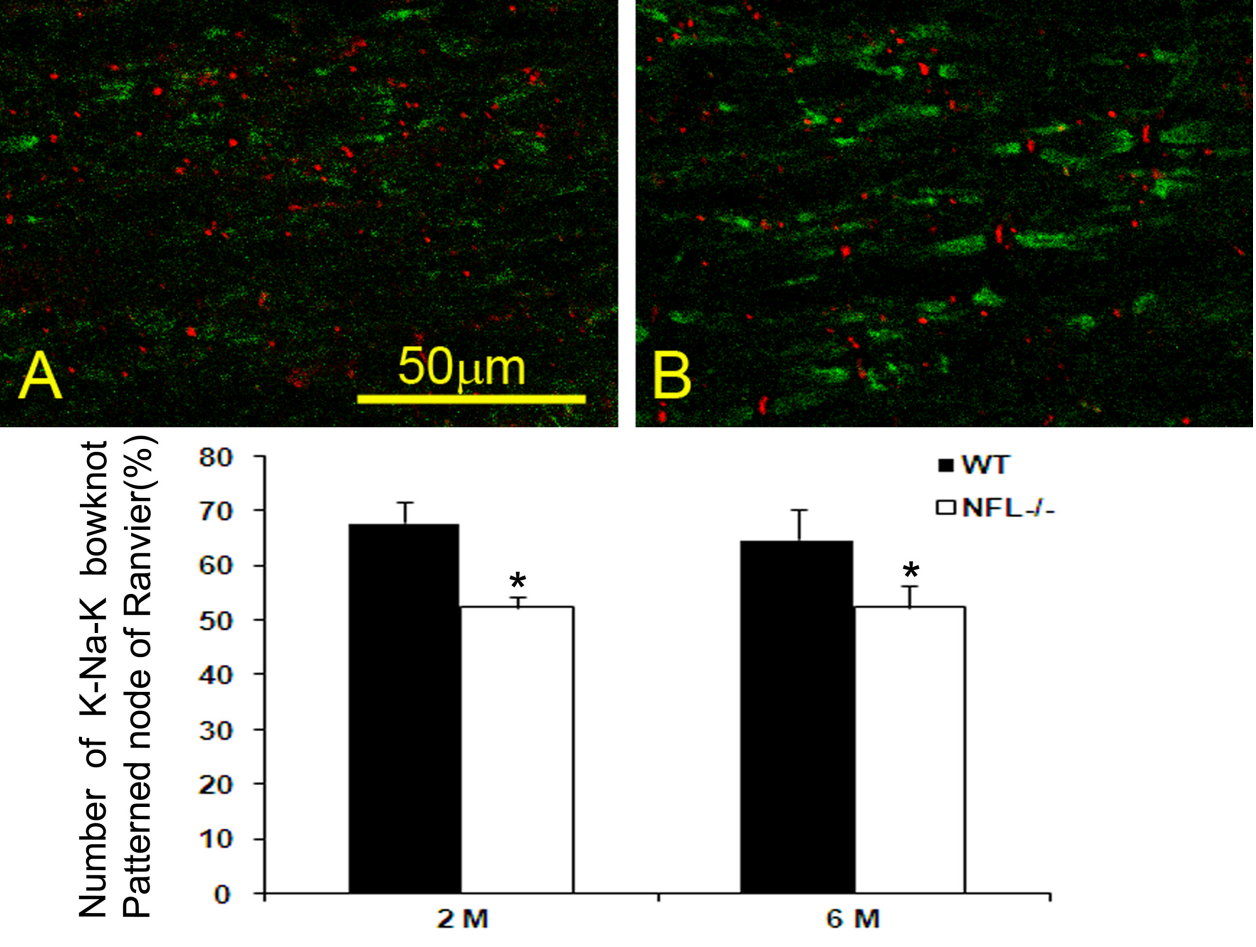
**Double labelling of Na^+ ^and K^+ ^channels**. Na^+ ^channels (Red) in the centre of the node and K^+ ^channels in both sides of the juxtaparanode (Green). In wild type mice (B), the double labelling appeared unique K-Na-K bowknot pattern while the percentage of the unique pattern decreased significantly in NFL-/- mice (A and bar chart, *p < 0.05). (A and B from mice at age of 2 months, N = 3 animals per group).

In the myelinated axon, Na^+ ^channels are located mainly in the node of Ranvier while the K^+ ^channels were found at the regions of juxtaparanode. Double labelling of Na^+ ^and K^+ ^channels shows a unique K-Na-K bowknot pattern in the wild type mice (Fig. 10B). From the over view of the photos, the bowknot shaped K-Na-K channels are more abundant and better organized in the spinal cord white matter of wild type mice compared with those of NFL-/- mice. The number of the K-Na-K patterned structures was significantly reduced in NFL-/- mice (Fig. 10, bar chart). There was no difference in wild type or NFL-/- mice among different age groups (*p *> 0.05).

### 4. NG-2 cell response

In the white matter region containing corticospinal tracts of the spinal cord, NG2 cells were noted to respond normally to axonal alterations in mice with disrupted NFL expression.

#### Loss of contact with the node of Ranvier

**Figure 11:**
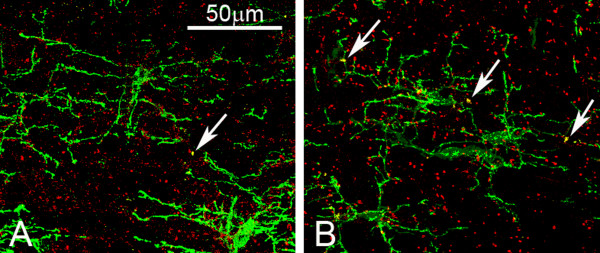
**Double labelling of Na^+^ channels and NG2 cells**. The micrographs show double labelling of Na channels (Red) and NG2 cells (Green). The number of yellow dots of double labelling was obviously reduced in the white matter of NFL-/- mice (A) in compared to that in wild type mice (B). (A and B from mice at age of 2 months, N = 3 animals per group).

Co-localization (yellow dots) of Na^+ ^channels (Red) and NG2 molecules (Green) was observed in the sections double stained for Na^+ ^channel and NG2 (Fig. 11). Co-localization was significantly lesser (*p *< 0.05) in NFL-/- mice (9.67 ± 2.08 at age of 2 months and 10.67 ± 3.51 at age of 6 months) when compared against wild type mice (17.67 ± 4.93 at age of 2 months and 20.00 ± 4.00 at age of 6 months), suggesting that the processes of NG2 cells might contact with the axon membrane at the node of Ranvier and that this connection might be lost upon targeted disruption of NFL expression in neurons.

#### Elongation and increase of NG2 cell processes

**Figure 12:**
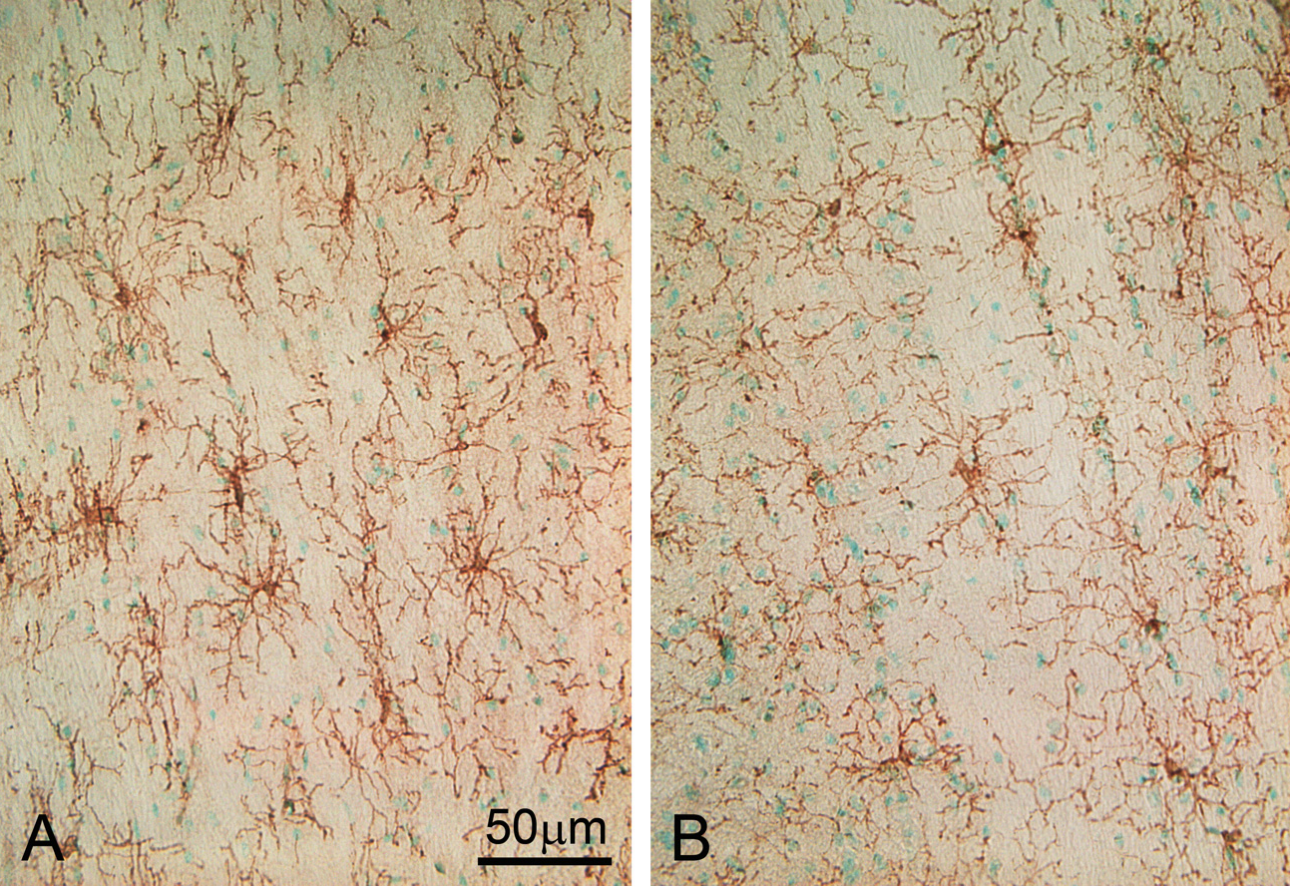
**Morphological changes of NG2 positive cells**. NG2 cells presented a small cell body and multiple fine processes. It appears that NG 2 cells in NFL-/- mice (A) have longer or more processes than those in wild type mice (B). (A and B from mice at age of 6 months).

Photos of NG2 cells are shown in Fig 12. The NG2 cellular processes in NFL-/- mice were longer and the cells had more branches (Fig. 12A) compared with those in wild type mice (Fig.12B). No significant difference (*p *> 0.05) was found in the number of NG2 cells in the white matter region containing corticospinal tract between NFL-/- (25.40 ± 2.61 at age of 2 months and 26.60 ± 2.30 at age of 6 months) and wild type mice (23.20 ± 3.12 at age of 2 months and 24.40 ± 4.00 at age of 6 months). The number of intersecting points between NG2 cell branches and 10 lines equally spanned in the view field was counted from the pictures. There were significantly more NG2 cellular processes crossing those fixed lines (*p *< 0.01) in the white matter of the spinal cord in NFL-/- mice (162.5 ± 8.81 at age of 2 months and 171.60 ± 10.53 at age of 6 months) compared against wild type mice (120.5 ± 15.61 at age of 2 months and 129 ± 8.93 at age of 6 months), suggesting that NG2 cells in NFL-/- mice had a larger number or longer processes.

### 5. No change in the expression of MAG and CNPase

**Figure 13:**
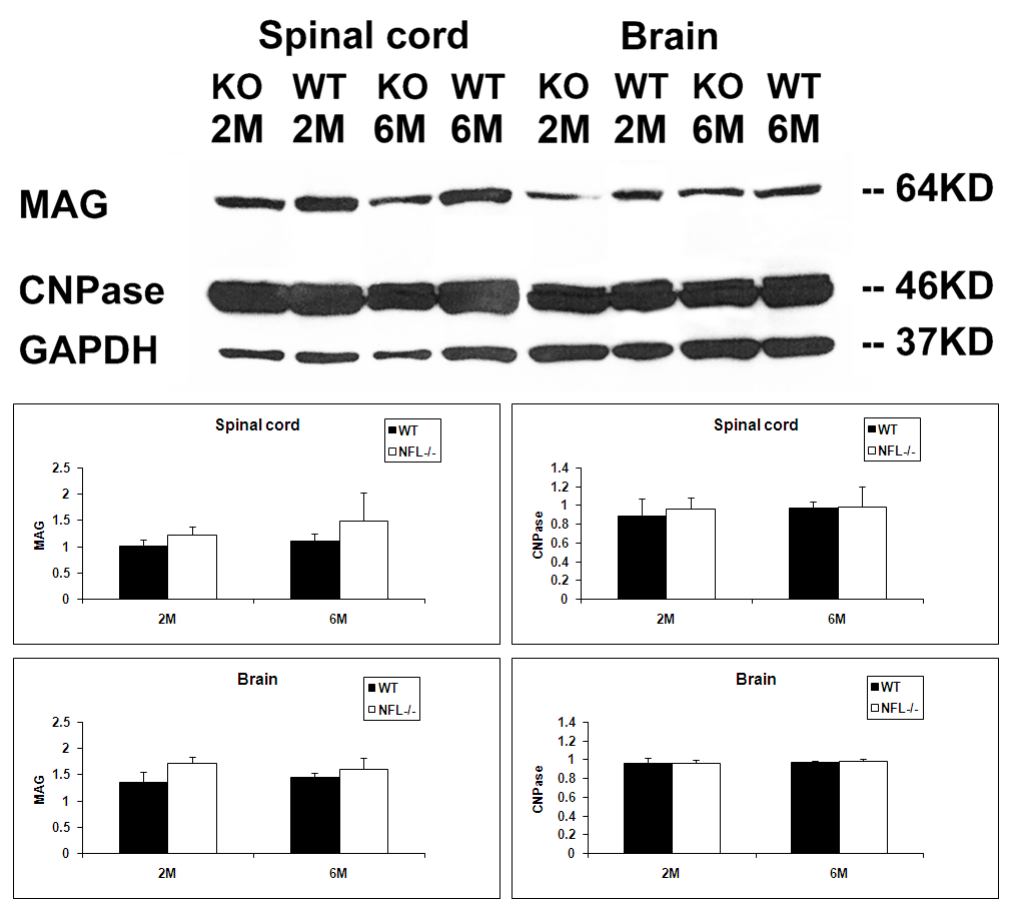
**Western blot of MAG and CNPase**. There was no significant difference in MAG or CNPase expression in brain cortex and spinal cord between wild type and NFL-/- mice.

Myelin associated glycoprotein (MAG) may play an important role in axonal reorganization and adhesion, intermembrane spacing, signal transduction during glial cell differentiation, regulation of neurite outgrowth, and maintenance of axon-myelin integrity; hence, we measured the expression of MAG protein using Western blot. The results showed no significant difference in the cortex of brain or spinal cord between C57BL/6 and NFL-/- mice (Fig. 13) at the early stage of neuropathogenesis in the NFL-/- mice. The level of CNPase, an oligodendrocyte marker, may decrease together with myelin reduction in various neurodegenerative diseases and experimental conditions. However, Western blot showed no significant alteration in CNPase expression in the brain or spinal cord when comparing wild type and NFL-/- mice at age of 2 or 6 months (Fig. 13), indicating that the amount of myelin was similar in both groups.

## Discussion

In this study, we used an animal model in which neuropathological progress was initiated by targeted disruption of NFL subunit expression in neurons. Morphological distortion and degeneration in myelinated axons were observed in the white matter region containing corticospinal tract in 2- and 6-month old NFL-/- mice. We have found previously that, until 6-month old, these early neuronal changes do not activate astrocytes or microglia. Instead, the environment has imposed an inhibitory effect on microglia [3]. However, NG2 cells at that earlier period of neuropathological development have the ability to sense and respond to the alterations in NFs of myelinated axons.

NFs may be a major determinant of axonal diameter, based on the correlation between NF contents in the axonal cross-sections and axonal calibres [18]. It was observed that small unmyelinated axons contain few NFs [19] and that some small neurons lack morphologically identifiable NFs [20,21]. This correlation persists during axonal degeneration and regeneration [22], and changes in NF transport correlate temporally with alterations in the calibre of axons in regenerating nerves [23]. In a study using a transgenic approach, NFL was confirmed as a major determinant of axonal calibre in the ventral root of lower motor neurons of the spinal cord [24]. In agreement with this observation, our earlier study has reported that the distribution of diameters of myelinated axons of upper motor neurons in the white matter of the spinal cord has shifted towards a smaller range in both 2- and 6-month old mice. This indicates that, after targeted disruption of expression of NFL, most NFH proteins cannot move into axons [3] resulting in an alteration in NF contents in myelinated axons and thereby inhibiting radial growth of the axons [24-28].

It is well accepted that the axon calibre controls the onset and rate of myelin formation, i.e., the thickness of the myelin sheath normally increases with axon calibre [29], although some reports suggested no apparent correlation between myelination and axonal size [30]. In our results, the G-ratio has decreased significantly at both ages of 2 and 6 months, suggesting a significant decrease in the diameter of axons and/or a significant increase in the thickness of myelin sheath. Further analyses showed that the alteration in NF contents in axons of NFL-/- mice has resulted in both significant decrease in axonal diameters and significant increase in the thickness of myelin sheath. The myelin sheath from a larger axon would now have to wrap around a smaller axon, resulting in an increase in the thickness of myelin sheath. More unloading loops in 2-month old mice also suggest more layer of myelin sheath wrapping around the axon. This implies that the alteration in NF contents in NFL-/- axons did not significantly change the amount of myelin synthesis as well as the processes of myelin wrapping. It is clear that signalling communication between the myelinating cells and neurons may not be determined by NF contents in axons.

In fact, our experimental results did not show any signs of oligodendrocytes actively responding to axonal alteration. CNPase (2', 3'-Cyclic Nucleotide 3'-Phosphodiesterase) is virtually only expressed by oligodendrocytes in the CNS and by Schwann cells in the PNS. CNPase activity is decreased in demyelinating diseases such as multiple sclerosis. Myelin-associated glycoprotein (MAG), a transmembrane glycoprotein, is selectively localized in periaxonal Schwann cell and oligodendrocyte membranes of myelin sheaths, suggesting that it functions in glia-axon interactions in both the PNS and CNS. Disruption of periaxonal myelin and loss of MAG is characterized by demyelination in rat spinal cord, which can be induced by injection of lipopolysaccharide [31]. However, in our observation, there was no obvious difference in the amount of CNPase and MAG, indicating that oligodendrocytes may not have responded to the alteration in axonal contents at early stage of development of NFL-/- mice.

While there was microglial inhibition, no obvious astrocyte and oligodendrocyte reactions in the spinal cord of NFL-/- mice at the age of 2 and 6 months were noted. The 4^th ^major non-neuronal cell type, NG2 cells, presented their ability to sense and respond to axonal changes at an earlier time window. Normally, the processes of NG2 expressing cells can closely contact nodes of Ranvier [5,32]. Some of this connection was lost as evidenced by the reduction in the number of NG2 and Na^+ ^channel double positive labelling in the NFL-/- neurons where profound redistribution of NFs occurred. Another sign of NG2 response was the elongation and increase of their processes. The purpose of this disconnection, process elongation and increase is unknown since the function of NG2 cells is not yet defined. The NG2 processes may provide a stable environment for axons and/or provide an inhibitory mechanism to prevent axonal sprouting at the node of Ranvier [32]. But the results from NG2 knockout mice showed that NG2 cells may be irrelevant for inhibition or promotion of axonal growth *in vivo *[33]. Therefore, the loss of connection between NG2 cells and axons might induce the elongation and increase of NG2 cell processes in an attempt to re-establish the connection to neurons and provide protection. However, the signal to attract NG2 cell processes to contact axons may decrease significantly after targeted disruption of NFL expression in neurons, resulting in a failure in the connection between NG2 cells and the nodes of Ranvier. It is also possible that NG2 cells may actively respond to alteration in NFL-/- axons and mediate further glial cell reaction in a later stage. Nevertheless, it is still a mystery about the role of early responding NG2 cells in the chain of glial reaction in neurodegenerative diseases.

## Conclusion

Our results demonstrated that the structural configuration determined by the NFL gene is important for the maintenance of normal morphology of myelinated axons. The alteration in axonal contents caused by disruption in NFL expression may not be able to result in severe demyelination but definitely leads to NG2 cell response. The NG2 cells might serve as an early sensor to deliver information of pathological changes in neurons to the local environment.

## Methods

### Animals

Two and 6 month old male NFL-/- mice with C57BL/6 background [24] (courtesy from Dr Julian, Canada) were housed five to a cage and acclimated to a 12 hour shift in light/dark cycle with free access to food and water. In total, 52 mice were used. For NFL-/- mice, five animals per time interval were used for immunohistochemistrical staining and 3 per time interval for electron microscopic study. Another five animals per time interval were employed for Western blot. The same number of C57BL/6J mice (Laboratory Animal Centre, Singapore) was used as the control. All procedures involving animals were according to guidelines of the Institutional Animal Care and Use Committee, National University of Singapore.

### Immunohistochemistry

Mice were perfused intra-aortically with ice-cold Ringer's solution (pH 7.4) and 2% PLP (pH 7.4). The brain and lumbar enlargement of the spinal cord were removed, postfixed in the same fixative for 4 hours at 4°C, and equilibrated in 30% sucrose overnight at 4°C. The coronal sections (20 μm) of the brain and transverse and longitudinal sections (20 μm) of the spinal cord were cryocut. Every 5^th ^section was collected on slides. Sections were blocked with 5% goat serum/PBS plus 0.1% Triton X-100 before being incubated with primary antibodies.

For DAB staining, the incubation of rabbit antibody against NG2 (1:2000, marker for NG2 cells) or rabbit antibody against NFH (1:1000, marker for neurons), over night at room temperature (RT) was followed by successive incubations with biotin-conjugated anti-rabbit secondary antibody (goat polyclonal, 1:200; Chemicon International, USA), ABC reagents (Vector Laboratories, USA), and SigmaFast DAB Peroxidase Substrate (Sigma-Aldrich, USA). Sections were counterstained with methyl green, before being dehydrated in graded ethanol, cleared in Histoclear, mounted with Vectashield mounting medium (Stem cell technology, USA) and then observed under a light microscope (Olympus BX51, Japan).

For immunofluorescent double labelling, rat antibody against potassium channel Kv1.2 (1:200, Sigma, USA) and mouse antibody against sodium channel (pan) clone K58/35 (1:200, Sigma, USA) were applied to sections for 2 hours at RT. Rabbit antibody against NG2 (1:200) combined with rat antibody against sodium channel (pan) clone K58/35 (1:200, Sigma, USA) were applied to sections for 2 hours at RT. Rat antibody against CD11b (1:1000, marker for microglia, BD Pharmingen, USA) was applied to spinal cord sections for 2 hours at RT. Then the FITC or Cy3-conjugated anti-rabbit or anti-rat secondary antibody (goat polyclonal, 1:200; Chemicon International, USA) was used and mounted with Vectashield mounting medium counterstaining. The slides were examined under a confocal microscope (Olympus LSM 510 Zeiss, Japan). Ten pictures from 10 sections of each animal were randomly taken from white matter with corticospinal tract of the spinal cord. In the sections of double labelling of Na^+ ^and K^+ ^channels, the number of intact unique K-Na-K labelling pattern was counted. In the sections of double labelling of NG2 and pan-Na channel, the number of positive double labelling was counted. The length of NG2 cell processes was also stereologically analyzed by number of the point where NG2 cell processes cross lines of square measuring units.

### Electron microscopic study

For conventional electron microscopy, the animals were perfused transcardially with Ringer's solution and then 2% paraformaldehyde containing 3% glutaraldehyde in 0.1 M phosphate-buffer. Transverse and longitudinal sections of the spinal cord were cut at 1 mm^3 ^and immersed in the same fixative for 4 hours at 4°C. Tissues were rinsed in 0.1 M phosphate-buffer (with 5% sucrose) with 3 changes at 10 minutes each. Post fixing of samples was done in 1% osmium tetroxide in 0.1 M phosphate-buffer (pH 7.4) for 2 hour at room temperature and dehydration in an ascending ethanol series at room temperarure. Tissue blocks were infiltrated in 100% acetone: resin (1:6) for overnight and three changes of pure resin at series of ascending temperature (40°C, 50°C, and 55°C) for 1 hour each. Samples were embedded in a pure araldite mixture at 60°C for 24 hours. Cross-section and longitudinal section of spinal cord blocks were cut into 99 nm thickness. Ultrathin sections were double stained in lead citrate and 3% uranyl acetate, and then were viewed in a Philips 120 Biotwin TEM (Fei company, USA).

For each fiber on cross-sections, the G ratio was calculated as d/D, where the d was the diameter of axon proper and the D were the outer diameter of the myelinated fiber. The g-ratio of myelianted fibers was determined in cross sections of spinal cord from NFL-/- mice and corresponding wild type mice. Randomly selected non-overlapping fields were photographed. Thickness of myelin sheath was calculated as half of the result of the outer diameter of the myelinated fibre minus the diameter of axon proper. Ten pictures from 2 sections of each animal were taken in either longitudinal or transverse section. Longitudinal sections of spinal cords from NFL-/- mice and wild type mice were employed to determine the number of oligodendrocyte loops and transverse sections axonal diameter, G-ratio, and thickness of myelin sheath. The measurements were taken under Leica Qfluoro System.

### Western blot

Protein extracts from the brain and spinal cord tissues were prepared. Total protein concentration was determined using the Bio-Rad protein assay (Bio-Rad Laboratories, USA). Western blot analysis of MAG and CNPase were carried out. Briefly, sample proteins were size separated through denaturing SDS-PAGE. An equal amount of protein for each sample was heated at 100°C for 5 minutes with an equivalent volume of sample buffer and loaded onto polyacrylamide gels. The proteins were electrotransferred to a polyvinylidene difluoride (PVDF) membrane in Tris-glycine-methanol buffer. The membrane was blocked for 1 hour at room temperature (RT) in a blocking solution mixture with 5% nonfat dry milk in 0.05% Tween 20 and TBS (TBST), pH 8.0. The membrane was washed with TBST on a shaker at RT for 3 times of 10 minutes and incubated overnight at 4°C with the following antibodies: rabbit anti-MAG (1:2000, Santacruz, USA) or mouse anti-CNPase (1:1500, Chemicon USA) in 1% bovine serum albumin in TBST. After repeated washings, the membrane was incubated with horseradish peroxidase-conjugated goat anti-mouse IgG (1:2000, Pierce, USA) or goat anti-rabbit IgG (1:2000, Pierce, USA) for 1 hour at RT (25°C). After washings, immunoreactivity was visualized using a chemiluminescent substrate (Supersignal West Pico, Pierce, USA). Loading controls were carried out by incubating the blots at 50°C for 30 minutes with a stripping buffer (100 mM 2-mercaptoethanol, 2% SDS and 62.5 mM Tris-hydrochloride, pH 6.8), followed by reprobing with a mouse monoclonal antibody to GAPDH (1:2500 in TBST, Abcam, USA) on a shaker in cold room (4°C) over night and horseradish peroxidase-conjugated anti-mouse IgG (1:2000 in TBST, Pierce, USA) at RT for 1 hours. ECL solution (Amersham) was added and developed for 1 minute. Exposed films containing blots were scanned and the densities of bands measured using Quantity One Version 4 software (Bio-Rad, USA). Densities of investigated bands were normalized against those of GAPDH and the mean ratios calculated.

### Statistical analysis

Data are expressed as the mean ± standard error. For comparison of differences among multiple groups the two-way ANOVA was used and for comparison of difference between two groups the t-test was used. Statistical significance was set at *p *< 0.05.

## Competing interests

The authors declare that they have no competing interests.

## Authors' contributions

YJW carried out the electronic microscopy and Western blot studies, participated in acquisition of data, performed the statistical analysis, and drafted the manuscript. YFT carried out the immunohistochemistry study, participated in acquisition of data, and performed the statistical analysis. ZCX and ZMB supervised YJW in experiments and participated in preparation of manuscript. BPH designed the study, supervised YJW and YFT in experiments and statistical analysis, and drafted the manuscript. All authors read and approved the final manuscript.
